# 基于机器学习与甲基化组学筛选*EGFR*突变肺腺癌奥希替尼原发性耐药关键基因*SORD*

**DOI:** 10.3779/j.issn.1009-3419.2025.102.46

**Published:** 2025-12-20

**Authors:** Guowei LIANG, Hongfeng WU, Chaoyi JIA, Penghu GAO, Zhanrui ZHANG, Chen CHEN, Yongwen LI, Hongyu LIU, Jun CHEN

**Affiliations:** ^1^832003 石河子，石河子大学医学院第一附属医院胸外科; ^1^Department ofThoracic Surgery, First Affiliated Hospital, School of Medicine, Shihezi University, Shihezi 832003, China; ^2^300052 天津，天津医科大学总医院胸部肿瘤中心肺部肿瘤外科; ^2^Department of Lung Cancer Surgery, Tianjin Medical University General Hospital, Tianjin 300052, China; ^3^300052 天津，天津医科大学总医院，天津市肺癌研究所，天津市肺癌转移与肿瘤微环境重点实验室; ^3^Tianjin Key Laboratory of Lung Cancer Metastasis and Tumor Microenvironment, Tianjin Lung Cancer Institute, Tianjin Medical University General Hospital, Tianjin 300052, China

**Keywords:** 肺肿瘤, *SORD*, EGFR-TKIs, 耐药, DNA甲基化, Lung neoplasms, SORD, EGFR-TKIs, Drug resistance, DNA methylation

## Abstract

**背景与目的:**

肺腺癌（lung adenocarcinoma, LUAD）是非小细胞肺癌中最常见的亚型，而表皮生长因子受体（epidermal growth factor receptor, *EGFR*）突变是其主要的分子驱动事件。尽管第三代酪氨酸激酶抑制剂（trrosine kinase inhibitors, TKIs）药物奥希替尼已成为该类患者的标准一线疗法，但耐药性的产生严重限制了患者的长期生存获益。证据表明，表观遗传重塑是导致耐药的重要非遗传机制。其中，DNA启动子区的高甲基化可通过沉默关键抑癌基因或代谢调节基因，协助肿瘤细胞逃避药物杀伤。本研究旨在通过机器学习和高通量筛选，挖掘调控奥希替尼敏感性的关键基因，并解析甲基化修饰在其调控奥希替尼原发性耐药中的作用。

**方法:**

基于癌症基因组图谱（The Cancer Genome Atlas, TCGA）-LUAD *EGFR*突变队列，应用oncoPredict模型预测奥希替尼的半数抑制浓度（half inhibitory concentration, IC_50_）。通过多组学关联分析，分别筛选与IC_50_显著相关的转录组与甲基化组差异基因。根据“高甲基化-低表达”负相关模式对两组学数据取交集，并应用*LASSO*回归与*Bootstrap*验证筛选出最稳健的核心基因。最后，在*EGFR*突变LUAD细胞系中，通过药物敏感性测定、去甲基化处理及基因过表达/敲低实验，验证核心基因对奥希替尼耐药、细胞增殖及凋亡的影响。

**结果:**

多组学分析与机器学习算法鉴定出*SORD*为核心候选基因，其关键位点（cg06424894）甲基化水平与mRNA表达呈显著负相关。体外实验显示，奥希替尼耐药株H1650呈*SORD*高甲基化与低表达特征，去甲基化药物处理可显著恢复其表达；敏感株则呈相反趋势。功能实验证实，在H1650耐药细胞中过表达*SORD*可逆转耐药，抑制细胞增殖并促进凋亡。相反，在H1975和PC9敏感细胞中敲低*SORD*，则显著诱导耐药，促进增殖并抑制凋亡。

**结论:**

*SORD*的甲基化状态或表达水平可望成为预测奥希替尼疗效的潜在生物标志物，靶向*SORD*相关通路可能为克服奥希替尼耐药提供新策略。

## Abstract (en)

**Background and objective:**

Lung adenocarcinoma (LUAD) is the most common subtype of non-small cell lung cancer (NSCLC), and epidermal growth factor receptor (*EGFR*) mutation is its primary molecular driver event. Although the third-generation tyrosine kinase inhibitors (TKIs) Osimertinib has become the standard first-line therapy for such patients, the development of drug resistance severely limits long-term survival benefits. However, increasing evidence suggests that epigenetic remodeling is a crucial non-genetic mechanism contributing to resistance. Specifically, hypermethylation of DNA promoter regions can assist tumor cells in evading drug cytotoxicity by silencing key tumor suppressor genes or metabolic regulatory genes. This study aims to identify key genes regulating Osimertinib sensitivity through machine learning and high-throughput screening, and to dissect the role of methylation modifications in regulating primary resistance to Osimertinib.

**Methods:**

Based on The Cancer Genome Atlas (TCGA)-LUAD *EGFR* mutation cohort, the oncoPredict model was applied to predict Osimertinib half inhibitory concentration (IC_50_) values. Through multi-omics association analysis, differentially expressed genes in the transcriptome and methylome significantly associated with IC_50_ were screened, respectively. The intersection of the two omics datasets was identified based on the "hypermethylation-low expression" negative correlation pattern, and *LASSO* regression with *Bootstrap* validation was applied to screen for the most robust core genes. Finally, in *EGFR*-mutant LUAD cell lines, the effects of the core gene on Osimertinib resistance, cell proliferation, and apoptosis were validated via drug sensitivity assays, demethylation treatment, and gene overexpression/knockdown experiments.

**Results:**

Multi-omics analysis and machine learning algorithms identified *SORD* as the core candidate gene, with the methylation level of its key site (cg06424894) showing a significant negative correlation with mRNA expression. *In vitro* experiments revealed that the Osimertinib-resistant cell line H1650 was characterized by *SORD* hypermethylation and low expression, and demethylation treatment significantly restored its expression; whereas sensitive strains showed the opposite trend. Functional experiments confirmed that overexpression of *SORD* in H1650 resistant cells could reverse resistance, inhibit cell proliferation, and promote apoptosis. Conversely, knockdown of *SORD* in sensitive H1975 and PC9 cells significantly induced resistance, promoted proliferation, and inhibited apoptosis.

**Conclusion:**

The methylation status or expression level of *SORD* holds promise as a potential biomarker for predicting Osimertinib efficacy, and targeting *SORD*-related pathways may provide new strategies for overcoming Osimertinib resistance.

肺腺癌（lung adenocarcinoma, LUAD）是非小细胞肺癌（non-small cell lung cancer, NSCLC）最主要的亚型，占所有肺癌病例的40%-50%，是全球癌症相关死亡的主要原因之一^[1]^。在LUAD患者中，表皮生长因子受体（epidermal growth factor receptor, *EGFR*）突变是一个关键的驱动基因，尤其在非吸烟的亚洲女性患者中，*EGFR*突变率高达50%-60%^[2]^。对于*EGFR*敏感突变（如外显子19缺失或L858R点突变）的NSCLC患者，第三代EGFR-酪氨酸激酶抑制剂（EGFR-tyrosine kinase inhibitors, EGFR-TKIs）奥希替尼已成为标准的一线治疗方案^[3]^。尽管奥希替尼显著改善了患者的无进展生存期和总生存期，但其疗效仍面临严峻挑战。这些挑战不仅包括几乎所有初始治疗敏感的患者在治疗1-2年内出现的获得性耐药，也包括一部分具有*EGFR*敏感突变的患者从治疗开始就表现出的原发性耐药^[4]^。目前，关于奥希替尼的获得性耐药机制研究已大量开展，C797S二级突变、间质上皮细胞转化因子（mesenchymal-epithelial transition factor, *MET*）扩增或突变以及旁路激活、组织学转化等是导致奥希替尼获得性耐药的重要机制^[5]^。然而，对于原发性耐药的机制，特别是基于表观遗传学调控在奥希替尼原发性耐药中的作用机制，目前的理解仍然不足。

DNA甲基化是一种重要的表观遗传修饰方式，这种修饰不改变DNA序列，却能稳定遗传并调控基因表达。在肿瘤中，启动子区异常高甲基化常导致抑癌基因沉默，进而影响DNA修复和凋亡通路^[6]^。近年研究^[7]^发现，特定基因的甲基化状态可直接介导EGFR-TKIs获得性耐药，正成为预测疗效和克服耐药的重要表观遗传标志物与潜在治疗靶点。此外，异常的DNA甲基化，如抑癌基因启动子区的高甲基化，可导致其表达沉默，进而影响药物敏感性，被认为是EGFR-TKIs原发性耐药的可能潜在机制之一^[8]^。

近年来，深度学习与机器学习算法为整合高通量多组学数据提供了有力工具，这尤其依赖于对大型公共数据库，如癌症基因组图谱（The Cancer Genome Atlas, TCGA）和肿瘤药物敏感性基因组学数据库2（Genomics of Drug Sensitivity in Cancer 2, GDSC2）等的挖掘和分析。oncoPredict等工具^[9]^则是利用这类数据，通过机器学习方法，建立样品对药物敏感性的预测工具。尽管oncoPredict等工具能有效预测药物敏感性，但对于耐药机制的解析仍然缺乏。此外，对于表观遗传调控（如甲基化）与药物敏感性之间的相互作用探讨仍不充分。

本研究通过整合TCGA-LUAD队列的转录组和甲基化数据，利用oncoPredict等模型量化伴有*EGFR*突变患者的奥希替尼的半数抑制浓度（half inhibitory concentration, IC_50_），筛选受DNA甲基化调控并可能导致*EGFR*突变LUAD患者原发性耐药的关键基因，并对筛选出的关键基因*SORD*在具有*EGFR*突变的LUAD细胞中的功能进行初步探讨，旨在揭示其调控LUAD细胞对奥希替尼敏感性的潜在机制，为识别新型耐药生物标志物和开发个性化治疗策略提供新的思路。

## 1 资料与方法

### 1.1 数据获取及基因突变分析

本研究中有关LUAD患者的数据来源于cBioPortal数据库（https://www.cbioportal.org）中处理好的LUAD数据[name: Lung Adenocarcinoma (TCGA, PanCancer Atlas), Reference: TCGA, Cell 2018]，总共纳入了566个LUAD样本，包括转录组数据以及相应的临床信息。

使用cBioPortal数据库获取TCGA-LUAD患者*EGFR*基因突变信息，突变包含点突变、缺失突变、插入突变等。

### 1.2 *EGFR*突变LUAD患者对奥希替尼药物敏感性的计算及药敏相关基因筛选

提取TCGA中具有*EGFR*突变的LUAD患者对应的RNA-seq数据，使用oncoPredict（v1.2）包并结合GDSC2数据库，得到每例患者对应的IC_50_。

构建多阶段筛选策略以精准筛选核心基因。首先，结合*Spearman*相关性分析与*Limma*模型，对转录组及甲基化数据进行初步过滤，筛选IC_50_相关基因。随后，应用带有10折交叉验证的*LASSO*回归进一步去除冗余，实现特征降维。为确保结果的稳健性，我们引入*Bootstrap*重抽样技术进行了10,000次迭代验证，最终保留在多次模拟中均表现稳定的关键基因。

### 1.3 细胞培养

细胞系H1650、H1975、HCC827、PC9购自中科院典藏生物资源保藏中心。这些细胞系在含有10%胎牛血清的DMEM（诺普赛，PM150210）培养基、37 °C和5% CO_2_的湿润培养箱中培养。

### 1.4 细胞凋亡检测

为评估细胞在奥希替尼压力下的凋亡情况，药物刺激48 h后，收集上清中的悬浮细胞及贴壁细胞，使用0.25%胰蛋白酶/EDTA，37 °C消化5 min，以冷PBS洗涤2次。随后将细胞在缓冲液中与FITC Annexin V和PI共同孵育25 min。最后，使用NovoCyte 2000R流式细胞仪进行分析并处理数据。

### 1.5 细胞增殖实验（CCK-8法）

首先，将待测细胞按3000个/100 µL的密度接种于96孔板中，每组设置至少3个复孔。接种后将培养板置于细胞培养箱中过夜以确保细胞完全贴壁，期间尽量避免晃动以保证细胞分布均匀。次日，弃除孔内原培养基，按预设浓度加入新鲜配制的药液处理48 h。处理结束后，吸除含药培养基，每孔加入100 µL含10% CCK-8的培养基。随后将培养板用锡纸包裹避光孵育1 h，最后利用酶标仪在450 nm波长下检测各孔的吸光度（OD值）。

### 1.6 细胞转染与RNA干扰

对于*SORD*基因的过表达，过表达质粒购自质粒与蛋白共享库，使用线性聚乙烯亚胺转染试剂（MW 40,000）按照说明书进行转染。对于*SORD*敲低，使用了2条针对人*SORD*基因的siRNA混合物。序列分别为：5’-CAGAAUCCCUGAUGUUAAU-3’； 5’-GAUCAUCGGUAAAGCACCU-3’。使用Lipofectamine 2000（Invitrogen）按照标准步骤将siRNA池转染至细胞。转染48 h后收集细胞进行后续分析。

### 1.7 荧光定量聚合酶链式反应（polymerase chain reaction, PCR）

使用胰蛋白酶消化并收集细胞，使用SPARKeasy Cell RNA快速提取试剂盒（Sparkjade，中国山东）提取总RNA。测量RNA浓度后，取2 µg总RNA，使用逆转录试剂盒（Takara，中国北京）将其逆转录为cDNA。荧光定量PCR在SLAN-96P全自动PCR分析仪上进行。所有基因的表达水平均以*GAPDH*作为内参进行归一化。本研究所使用的*SORD*引物序列如下：F：5’-GCCGATACAATCTGTCACCTTCC-3’，R：5’-CGCCTTCCTCAAAGGTGACATTG-3’。

### 1.8 DNA亚硫酸氢盐转化及甲基化特异性PCR引物设计

提取各组样本的基因组DNA，使用DNA亚硫酸氢盐转化试剂盒（碧云天，编号：D0068S）对DNA进行转化处理。转化后的DNA存储于-80 ^o^C。针对*SORD*基因启动子区域的CpG岛，利用MethPrimer在线工具（http://www.urogene.org/methprimer/）设计甲基化特异性引物（M）和非甲基化特异性引物（U）。引物序列如下：*SORD*-M-L：5’-TATTTAAGTTTTAGTAATGGCGGAC-3’，*SORD*-M-R：5’-TATACCTAACTCAATAAATCCCACG-3’；*SORD*-U-L：5’-TTTAAGTTTTAGTAATGGTGGATGT-3’，*SORD*-U-R：5’-TACCTAACTCAATAAATCCCACACC-3’。采用荧光定量PCR法检测各组样本的甲基化水平。根据甲基化引物与非甲基化引物扩增所得Ct值（分别为Ct_M和Ct_U），按如下公式计算各样品中*SORD*基因的相对甲基化率：[2^-Ct_M^/(2^-Ct_M^+2^-Ct_U^)]×100%。

### 1.9 统计分析

所有统计分析均在R语言环境（v4.3.3）中进行。数据表示为均数±标准差（Mean±SD），两组间差异比较采用双尾非配对*Student's t*检验。*P*<0.05被认为差异具有统计学意义。

## 2 结果

### 2.1 基于机器学习预测TCGA-LUAD队列中*EGFR*突变患者的奥希替尼IC_50_

为预测LUAD患者对奥希替尼的敏感性，本研究利用cBioPortal平台对TCGA-LUAD数据集中的566例原始病例进行分析。以*EGFR*突变为筛选标准，共鉴定出79例携带*EGFR*突变的患者，其*EGFR*突变以经典激活突变为主，其中L858R点突变20例（23.3%）、外显子19 E746_A750del缺失14例（16.3%）；伴随或独立*EGFR*扩增21例（24.4%）。其余为少见激活突变（L861Q 3例、G719A/C 3例、S768I 2例）及罕见插入/缺失（E709_T710delinsD 3例、L747-T751del 2例、L747_A750delinsP 2例、A767_V769dup 1例、H773dup 1例、K754_I759del 1例、T751_I759delinsN 1例），并检测到耐药相关T790M 2例。余为散发突变或意义未明变异。

采用这79例样本的RNA-seq数据作为机器学习模型的分子层面输入信息。进一步选用oncoPredict作为核心计算算法，并以GDSC2作为模型预测的参考标准，通过oncoPredict计算，获取全部79例*EGFR*突变患者对奥希替尼的IC_50_预测值。结果显示，39例样本的IC_50_值高度密集于2.00-5.00 μmol/L区间，该群体涵盖了大部分L858R（10例）及E746_A750del（5例）突变样本；极敏感区间（<0.68 μmol/L）则富集了K754I、759del等突变。尽管L858R和E746_A750del通常被视为奥希替尼的经典敏感突变，但我们的预测结果显示，除L833F/L861Q等非经典突变外，仍有4例L858R和2例E746_A750del样本IC_50_落入高IC_50_（>10.00 μmol/L）抗性区间。随后，我们对IC_50_值进行了log_2_转换以利于后续统计分析。

### 2.2 结合RNA-seq与TCGA甲基化数据筛选药敏相关基因

为了进一步挖掘与奥希替尼IC_50_相关的关键基因，首先，我们提取患者的RNA-seq数据与奥希替尼IC_50_值进行相关性分析，共筛选出1173个与IC_50_显著相关的基因，其中呈正相关的基因1100个，呈负相关的基因73个。

同样的，我们对79例患者中70例拥有DNA甲基化数据的患者样本，采用类似的相关性筛选标准，分析基因甲基化水平与IC_50_的相关性。此分析共获得2652个基因甲基化水平与IC_50_显著相关的基因，其中正相关基因118个（甲基化水平越高，患者对应的奥希替尼IC_50_值越高，提示耐药相关），负相关基因2534个（甲基化水平越高，IC_50_值越低）。

为了进一步锁定受甲基化调控且与IC_50_显著相关的核心基因，基于表观遗传学中“DNA甲基化水平与基因表达呈负相关”的机制，进行筛选。具体筛选策略如下：首先，取DNA甲基化分析中与IC_50_显著正相关的“高甲基化基因”与转录组分析中与IC_50_显著负相关的“低表达基因”的交集，最终获得36个高甲基化且低表达且与IC_50_显著相关的候选基因（附图1， http://www.lungca.org/files/2025s192s1.pdf）。

### 2.3 基于*LASSO*回归筛选奥希替尼原发性耐药相关高甲基化基因

我们进一步应用*LASSO*回归模型，对上述36个高甲基化且低表达的基因进行特征筛选。结果显示，所构建的预测模型对药物敏感性（log_2_(IC_50_)）具有良好的预测性能，预测值与实际值的*Pearson*相关系数为0.738，决定系数为0.544。为进一步验证模型的稳健性并挖掘关键基因，我们采用了10,000次*Bootstrap*重抽样策略。结果显示，12个基因的入选频率超过50%，表明它们是与IC_50_强相关的稳定特征。这些基因包括*SORD*、*NEURL*3、*FIGN*、*ZNF280B*、*LANCL2*、*LYPD6*、*WDFY2*、*ZNF418*、*BEND3*、*TIAM1*、*POM121*和*RNF220*。值得注意的是，排名靠前的基因在功能上均高度富集于癌症进展的关键通路：*SORD*是代谢重编程的关键酶，*FIGN*是调控微管动态和有丝分裂的重要蛋白，*ZNF280B*作为转录因子参与p53信号通路的调控，而*TIAM1*则介导Rac1信号通路以影响细胞迁移。在所有稳定入选的基因中，代谢酶*SORD*的选择频率最高（0.7859），提示*SORD*是与药物敏感性（IC_50_）关联最密切的核心候选基因（附图2，http://www.lungca.org/files/2025s192s2.pdf）。

### 2.4 *SORD*基因表达受甲基化调控

为确认*SORD*为受甲基化调控基因，我们通过cBioPortal平台工具分析TCGA-LUAD甲基化数据集。结果显示，*SORD*关键甲基化位点甲基化水平与其mRNA表达呈显著负相关，特别是cg06424894位点（TSS1500）相关性尤为显著（*Spearman: r*=-0.21, *P*=1.567×10^-6^; *Pearson: r*=-0.17, *P*=7.462×10^-5^）（图1A、1B）。同时，TCGA样本分析显示*SORD*基因突变频率极低，排除了基因突变作为影响其表达水平的因素。

**图 1:**
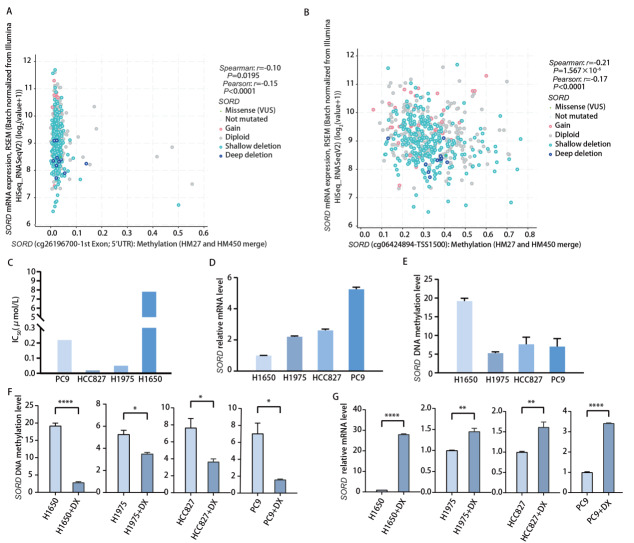
候选基因SORD的表达验证及其与甲基化的相关性分析。A：SORD基因甲基化位点（cg26196700-1st Exon; 5'UTR）与其mRNA表达水平的相关性散点图；B：SORD基因甲基化位点（cg06424894-TSS1500）与其mRNA表达水平的相关性散点图；C：H1650、HCC827、H1975和PC9细胞系对奥希替尼的药物敏感性检测（IC_50_）；D：不同细胞系中SORD基因的基础mRNA表达水平；E：不同细胞系中SORD基因的基础甲基化水平；F：地西他滨处理显著降低了不同细胞系中SORD基因的甲基化水平；G：地西他滨处理引起的甲基化水平降低显著影响了不同细胞系中SORD mRNA的表达变化。

为了在体外水平进行验证，我们采用4种LUAD细胞株（H1650、H1975、HCC827、PC9），这4种LUAD细胞株均具有*EGFR*突变，其中H1650、PC9、HCC827均携带*EGFR*外显子19缺失突变，H1975携带*EGFR* L858R与T790M双突变。我们首先评估了4种细胞株的奥希替尼敏感性。结果显示，H1650细胞对奥希替尼表现为原发性耐药（IC_50_约7.8 μmol/L），而其他3种细胞（H1975、HCC827、PC9）则均为敏感细胞系（IC_50 _≤0.22 μmol/L）（图1C）。

*SORD*基因的甲基化特异性PCR检测显示，耐药的H1650细胞呈现出*SORD*基因高甲基化状态，mRNA表达水平低；与此相反，3种敏感细胞系则均表现为低甲基化状态，*SORD* mRNA表达水平高（图1D、1E）。

4种细胞株进一步采用30 μmol/L去甲基化药物地西他滨处理48 h。结果显示，地西他滨可显著提升其mRNA表达，H1650细胞*SORD* mRNA水平相较于原始表达提升20倍以上（图1F、1G），支持*SORD*是一个受甲基化调控的基因。

### 2.5 *SORD*基因表达可影响*EGFR*突变LUAD细胞对奥希替尼的敏感性

为验证*SORD*基因对奥希替尼疗效的直接影响，本研究进行了细胞表型实验。首先，在耐药细胞系H1650中过表达*SORD*基因（图2A），IC_50_测定结果表明，*SORD*过表达显著逆转了H1650细胞的耐药性，IC_50_值从7.8 μmol/L降低至5.8 μmol/L（图2B）。在5 μmol/L奥希替尼处理下，CCK8实验显示*SORD*过表达显著抑制了细胞的增殖能力（与对照组相比，*P*=5.60×10^-3^）（图2C、2D）。

**图 2:**
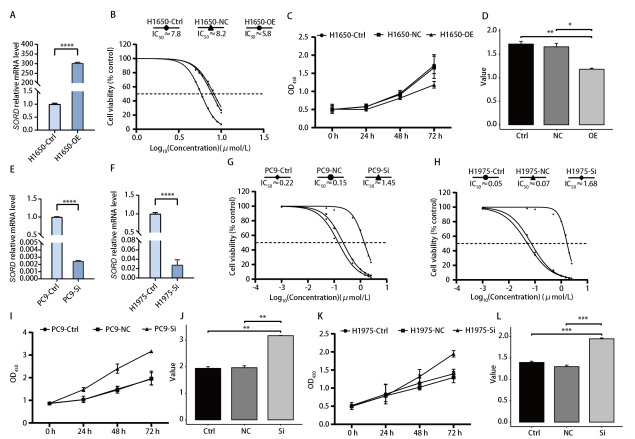
调控SORD基因表达对非小细胞肺癌细胞奥希替尼耐药性的影响。A：通过质粒转染在H1650细胞中过表达SORD基因；B：过表达SORD基因后H1650细胞对奥希替尼敏感性的变化曲线；C、D：评估过表达SORD基因后细胞在药物作用下的增殖能力变化；E、F：通过siRNA转染在PC9和H1975细胞中敲低SORD基因的表达；G、H：敲低SORD基因后PC9和H1975细胞对奥希替尼敏感性的变化曲线；I-L：敲低SORD基因后细胞在药物作用下的增殖能力变化。

同时，在敏感细胞系H1975和PC9中敲低*SORD*基因（图2E、2F），结果显示，*SORD*敲低显著增加细胞耐药性：PC9细胞系IC_50_从0.22 μmol/L升至1.45 μmol/L，H1975细胞系IC_50_从0.05 μmol/L升至1.68 μmol/L（图2G、2H）。在0.05 μmol/L低浓度奥希替尼作用下，CCK8实验亦表明*SORD*敲低显著提升了细胞的增殖能力（PC9组*P*=0.0022，H1975组*P*=0.00018）（图2I-2L）。

为进一步探究*SORD*对细胞凋亡的影响，我们在H1650过表达细胞中检测了5 μmol/L奥希替尼处理48 h后的凋亡情况。结果表明，*SORD*过表达显著提升了细胞凋亡率（*P*=1.70×10^-4^）（图3A、3B）。同样，在H1975-Si *SORD*和PC9-Si *SORD*细胞中，0.05 μmol/L奥希替尼处理48 h后，SORD敲低显著抑制了细胞凋亡（PC9组*P*=1.70×10^-2^，H1975组*P*=8.1×10^-6^）（图3C-3F）。

**图 3:**
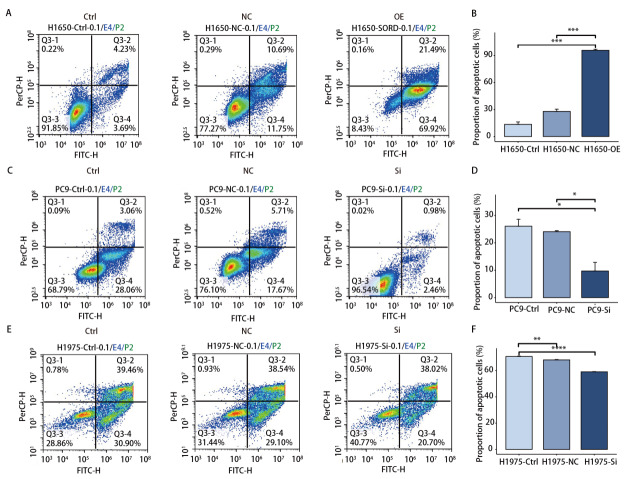
干扰SORD基因表达对奥希替尼诱导的细胞凋亡的影响。A、B：H1650细胞过表达SORD基因后，流式细胞术检测在奥希替尼处理下的细胞凋亡情况；C、D：PC9细胞敲低SORD基因后，流式细胞术检测在奥希替尼处理下的细胞凋亡情况；E、F：H1975细胞敲低SORD基因后，流式细胞术检测在奥希替尼处理下的细胞凋亡情况。

## 3 讨论

奥希替尼虽显著改善了*EGFR*突变LUAD患者的预后^[10]^，但原发性耐药仍是主要的临床瓶颈。DNA甲基化作为关键的表观遗传修饰，已被证实可通过沉默抑癌基因或激活耐药通路影响EGFR-TKIs疗效^[11]^，并促进耐药相关通路的激活^[12]^。因此，挖掘受甲基化调控的关键耐药节点，对于开发新型生物标志物及增敏策略具有重要意义。

与既往研究主要依赖细胞系模型或单一组学数据不同，本研究创新性地构建了“药物反应预测-多组学交互-机器学习筛选”的整合分析框架。首先，利用oncoPredict模型在TCGA临床队列中克服了药物反应数据缺失的难题；其次，通过转录组与甲基化组的交集分析，精准锁定了“表观遗传沉默”特征基因；最后，应用*LASSO*回归与*Bootstrap*重抽样验证，确保了筛选结果的稳健性与可靠性。这一严谨的筛选流程使我们最终锁定了*SORD*作为与奥希替尼耐药最相关的核心基因，避免了单一分析方法可能产生的假阳性偏差。

SORD编码的山梨醇脱氢酶是多元醇通路的关键限速酶^[13]^，该通路通过醛糖还原酶AKR1B1与SORD催化的两步酶促反应，实现葡萄糖向果糖的转化^[14]^。因此，现有研究多聚焦于其在糖尿病并发症中的病理作用^[15]^以及周围神经病变^[16]^，而该基因在肺癌耐药进程中的表观遗传调控机制则鲜有报道。本研究结果显示，*SORD*启动子区域的过度甲基化与其mRNA表达沉默呈显著负相关，这一发现在耐药细胞株H1650中得到了体外验证。 这一结果直接提示，DNA甲基化是导致*SORD*在耐药株中失活的主要机制。功能实验进一步证实，*SORD*并非单纯的“旁观者”，而是耐药表型的“驱动者”：在耐药株中恢复*SORD*表达可显著逆转耐药并诱导凋亡，而在敏感株中敲低*SORD*则重现了耐药表型。这表明*SORD*的缺失可能通过破坏细胞内的山梨醇/果糖代谢平衡，改变细胞氧化还原状态或能量代谢流，从而赋予癌细胞在药物压力下的生存优势^[17]^。与*METTL3*介导的m6A修饰等^[18]^其他机制相比，本研究突出了DNA甲基化驱动的特定代谢基因沉默在奥希替尼耐药中的独特作用。

总之，本研究通过多组学整合分析与机器学习算法，不仅筛选了*SORD*甲基化在奥希替尼耐药中的调控价值，也为开发广谱性TKIs耐药逆转策略提供了新思路。在NSCLC治疗已进入分子分型指导的精准时代，靶向表观遗传的疗法正成为克服靶向治疗耐药的重要方向，而本研究为这一领域的药物研发提供了新的潜在靶点。
